# The Hidden Threat of Pharmaceuticals: Ketoprofen Degradation and Toxicity to Non-Target Organisms

**DOI:** 10.3390/molecules31060949

**Published:** 2026-03-12

**Authors:** Paweł Solski, Urszula Guzik, Danuta Wojcieszyńska

**Affiliations:** Institute of Biology, Biotechnology and Environmental Protection, Faculty of Natural Science, University of Silesia in Katowice, Jagiellońska 28, 40-032 Katowice, Poland; pawelsolski12345@wp.pl (P.S.); danuta.wojcieszynska@us.edu.pl (D.W.)

**Keywords:** ketoprofen, biodegradation, toxicity

## Abstract

Ketoprofen is a widely prescribed non-steroidal anti-inflammatory drug whose extensive global use, combined with limited biodegradability, has led to its increasing detection as a micropollutant in aquatic and terrestrial environments. Incomplete removal during wastewater treatment results in its continuous release into surface waters and soils, creating conditions for chronic, low-dose exposure of non-target organisms. This review synthesizes current knowledge on the physicochemical characteristics of ketoprofen, its mechanism of action, environmental occurrence, degradation pathways, and ecotoxicological effects. Particular emphasis is placed on biological and photochemical transformation processes that influence ketoprofen persistence and toxicity. While the acute toxicity of ketoprofen has been relatively well documented, data on chronic toxicity remain scarce, despite growing evidence that long-term exposure may pose significant ecological risks. Studies indicate that low environmental concentrations can induce hormetic responses in animals and plants, whereas higher levels may cause cellular damage associated with oxidative stress, affecting organisms ranging from microorganisms to vertebrates and vascular plants. By integrating available data on ketoprofen degradation and toxicity, this review highlights critical knowledge gaps regarding its chronic ecotoxicity and underscores the need for systematic environmental monitoring and the development of effective degradation strategies to mitigate risks to non-target organisms.

## 1. Introduction

Ketoprofen is one of the most commonly used anti-inflammatory drugs. Due to its widespread use and relatively low biodegradability, it is increasingly found in the environment as a micropollutant. It is a highly potent and persistent anti-inflammatory, analgesic, and antipyretic drug, derived from propanoic acid. It is widely used in over 80 countries worldwide to treat moderate pain and swelling associated with rheumatoid arthritis, osteoarthritis, and other inflammatory diseases. Furthermore, rapid absorption, easy metabolism, and rapid crossing of the blood–brain barrier are features that contribute to its widespread use and popularity [1]. Frequent use of this drug results in its presence in treated wastewater and surface waters, as ketoprofen is incompletely removed during wastewater treatment. In addition to its occurrence in aquatic environments, it can also be found in soil due to irrigation with treated wastewater [2]. Although only a few reports indicate a risk of toxic effects associated with the presence of ketoprofen in environmental samples, the ecological risk is most likely related to the impact of long-term exposure to low doses. Data on the chronic toxicity of ketoprofen are currently very limited [1].

Toxicity is a characteristic of chemical compounds defined as an undesirable, direct effect on the organism, caused by chemical or physicochemical reactions between a given chemical compound and the biological system of the organism into which it has entered. Factors influencing toxicity include dose, frequency, and duration of exposure, and route of administration. Toxicity observed after a single exposure often differs from that observed after repeated exposures. Long-term effects are usually associated with chronic exposure [3,4]. Acute toxicity, or harmful effects occurring within a defined period of time after a single exposure to a substance, is the subject of numerous studies on the toxicity of drugs, including ketoprofen. Determining acute toxicity is usually the first step in assessing a compound’s toxic properties using a bioassay. It provides information on the potential health risks associated with short-term exposure to a chemical. Generally, the strength or potency of an agent or stimulus (toxin) is determined by the response of the test organism [5]. Substances with an LD_50_ (Lethal Dose 50%) below 5 mg/kg are classified as highly toxic, while substances with an LD_50_ above 15,000 mg/kg are considered relatively harmless [6]. Chronic toxicity, which is much more challenging to analyse and less frequently studied, occurs due to exposure to a toxic agent over many months or years [7]. Chronic toxicity tests involve exposing an organism to a test substance at least five concentrations throughout the organism’s lifetime. Data obtained from such tests allow estimation of the lowest observed effect concentration (LOEC) and the no observed effect concentration (NOEC), which are widely used in the statistical analysis of chronic ecotoxicity data [8]. In the case of environmental toxicity, chronic toxicity towards organisms accidentally exposed to the toxin is particularly important. This publication is particularly important because it addresses the current—based on the latest research—yet still poorly understood problem of ketoprofen’s presence in the environment, pointing to significant gaps in knowledge regarding its chronic ecotoxicity. One key issue is the lack of systematic analysis of intermediates formed during both the physicochemical and biological degradation of ketoprofen. This deficiency is particularly significant because even fewer attempts are made to link identified intermediates with their potential toxicity. Consequently, current knowledge does not allow for reliable assessment of the ecological risk posed by ketoprofen in the environment. This paper also highlights methodological shortcomings, emphasizing the lack of studies conducted in situ and involving complex, numerous communities of organisms. Furthermore, it is indicated that—with few exceptions—there is a lack of information on the synergistic effects of ketoprofen with other compounds, and that its potential hormetic effects on organisms are still insufficiently considered.

## 2. Characteristics of Ketoprofen and Its Action Mechanism

Ketoprofen (2-(3-benzoylphenyl)propanoic acid) was first synthesised in 1968 and introduced to the pharmaceutical market in France and the United Kingdom in 1973 [9]. It is a bicyclic compound belonging to the class of 2-aryl propanoic acids, commonly referred to as profens. Ketoprofen exhibits poor aqueous solubility; however, its solubility is strongly influenced by environmental pH and the presence of surfactants such as sodium lauryl sulfate. The highest solubility has been observed in polar alcoholic solvents, including methanol, ethanol, isopropanol, and butanol [10].

Due to its limited solubility, increasing attention has been devoted to developing ketoprofen formulations with improved bioavailability. To this end, ketoprofen has been introduced in various forms, including the lysine salt, prodrug derivatives such as the ketoprofen–dextran ester, microencapsulated ketoprofen elixirs, and cyclodextrin inclusion complexes [11].

Ketoprofen is a chiral molecule that exists as two enantiomers, (S) and (R), and is most commonly administered as a racemic mixture. Its crystal structure consists of heterochiral RS dimers stabilised by hydrogen bonding between the carboxyl groups of the two enantiomers [10,12,13]. The enantiomers of ketoprofen exhibit distinct pharmacological properties. The (S)-enantiomer, known as dexketoprofen, is primarily responsible for the anti-inflammatory activity characteristic of nonsteroidal anti-inflammatory drugs (NSAIDs). In contrast, the (R)-enantiomer, despite being a weak cyclooxygenase inhibitor, demonstrates significant analgesic activity in humans. It is also used in dental formulations, such as toothpaste, to prevent periodontal diseases [10].

All profens are capable of undergoing chiral inversion; however, in humans, this process occurs predominantly for ibuprofen, fenoprofen, and benoxaprofen. In the case of ketoprofen, only a limited degree of inversion from the (R)- to the (S)-enantiomer has been observed [14]. The minimal extent of chiral inversion suggests that ketoprofen may exert its pharmacological effects through both cyclooxygenase-dependent and cyclooxygenase-independent mechanisms [15].

The pharmacological activity of ketoprofen is primarily based on the inhibition of arachidonic acid metabolism through nonselective suppression of cyclooxygenase (COX) isoenzymes. Cyclooxygenases are bifunctional enzymes that catalyse two sequential reactions in spatially distinct yet mechanistically coupled active sites: the double dioxygenation of arachidonic acid—released from membrane phospholipids via enzymatic activation of phospholipase A_2_—to prostaglandin G_2_ (PGG_2_), followed by the reduction of PGG_2_ to prostaglandin H_2_ (PGH_2_). Oxidation of arachidonic acid occurs at the cyclooxygenase active site, whereas the reduction of PGG_2_ takes place at the peroxidase active site. Access to the COX active site is provided by a long, hydrophobic channel that extends deep into the catalytic domain of the enzyme. Upon binding within the active site, arachidonic acid forms ionic interactions with the guanidinium group of Arg120 and hydrogen bonds with Tyr355, interactions that are essential for its correct positioning relative to the enzyme’s catalytic machinery [16,17].

**Figure 1:**
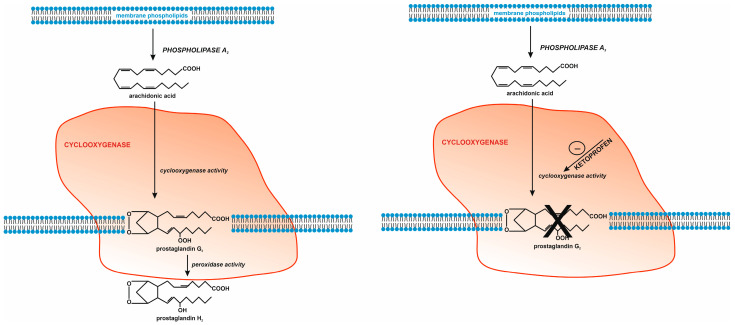
Ketoprofen’s mechanism of action involves competitive inhibition of the cyclooxygenase active site. Ketoprofen binds to the enzyme’s catalytic channel, occupying the space reserved for arachidonic acid and limiting its access to the catalytic center, thereby inhibiting prostaglandin biosynthesis.

The mechanism of action of ketoprofen involves a competitive blockade of the cyclooxygenase active site (Figure 1). By occupying the binding space normally reserved for arachidonic acid, ketoprofen restricts substrate access to the catalytic centre of COX, thereby inhibiting the biosynthesis of prostaglandins responsible for pain, fever, and inflammatory responses [18]. Ketoprofen binds within the catalytic channel of COX, a property characteristic of classical nonsteroidal anti-inflammatory drugs [19]. A key anchoring element of the ligand within the enzyme active site is the carboxyl group, which forms specific hydrogen bonds and electrostatic interactions with the conserved Arg120 residue located at the entrance of the active channel, as well as hydrogen bonds with Tyr355. These interactions ensure proper orientation of the ketoprofen molecule within the active site and stabilise the enzyme–inhibitor complex [18,19].

The aromatic moiety of ketoprofen penetrates deeply into the hydrophobic catalytic channel of COX, where it is stabilised by numerous hydrophobic and van der Waals interactions with amino acid residues forming the enzyme pocket, including Val349, Leu352, Phe381, Tyr385, Trp387, and Phe518. These interactions enhance the inhibitor’s affinity for the enzyme, effectively limiting substrate access to the catalytic centre and suppressing cyclooxygenase activity [20].

In contrast to aspirin, ketoprofen does not acetylate enzyme residues and therefore acts as a reversible inhibitor. However, its metabolite, in the form of the ketoprofen-CoA thioester (KTP–CoA), is a selective, irreversible inhibitor of COX-2, and its acylation of the enzyme is responsible for the sustained inhibition of this isoenzyme. The presence of this metabolite likely accounts for the greater COX-2 selectivity of ketoprofen than previously estimated based solely on data obtained for the parent compound [15,17].

Ketoprofen is considered one of the most potent cyclooxygenase inhibitors among classical nonsteroidal anti-inflammatory drugs. In studies evaluating the inhibition of prostaglandin synthesis in isolated guinea pig lungs, racemic ketoprofen exhibited approximately 6-fold greater inhibitory activity than naproxen. Moreover, its potency was reported to be 800–1500 times higher than that of ibuprofen, acetylsalicylic acid, and phenylbutazone [21].

Ketoprofen displays favourable pharmacokinetic properties, including efficient distribution to the central nervous system. By crossing the blood–brain barrier, it inhibits prostaglandin synthesis in the hypothalamus, thereby contributing to its analgesic and antipyretic effects [9]. In addition to inhibiting cyclooxygenase, ketoprofen has been shown to suppress lipoxygenase activity, an enzyme involved in the alternative pathway of arachidonic acid metabolism that leads to leukotriene synthesis. This pathway operates primarily in leukocytes, and its products play a crucial role in the initiation and maintenance of inflammatory responses by promoting leukocyte migration and activation. Inhibition of leukotriene biosynthesis by ketoprofen may therefore attenuate inflammation associated with cellular immune responses and potentially limit cartilage degradation in rheumatoid arthritis.

Furthermore, ketoprofen inhibits bradykinin activity, a key mediator of pain and inflammation. It also stabilises lysosomal membranes, protecting them from osmotic damage and preventing the release of hydrolytic enzymes that could otherwise exacerbate inflammatory processes [21,22].

Beyond its established analgesic and anti-inflammatory indications, ketoprofen has demonstrated therapeutic potential in various additional clinical contexts. These include the treatment and prevention of gastrointestinal tract injury, management of non-alcoholic fatty liver disease, lymphedema, and epileptic seizures in humans. Moreover, ketoprofen has been associated with antidepressant and anxiolytic effects, as well as potential anti-allergic activity, suggesting a pleiotropic pharmacological profile [9].

**Figure 2:**
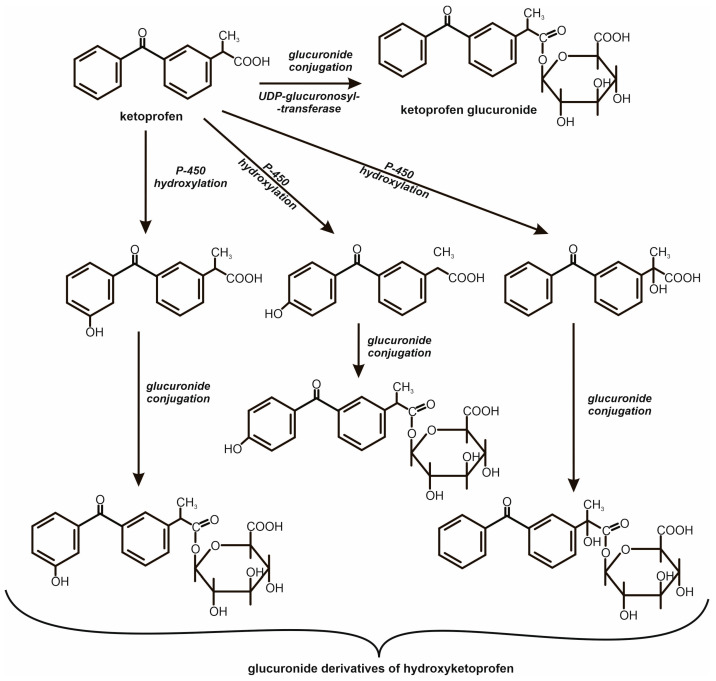
Biotransformation of ketoprofen in the human body via hydroxylation and glucuronic acid conjugation reactions [23].

Ketoprofen undergoes biotransformation in the liver of vertebrates through several metabolic pathways, including primarily glucuronidation, as well as benzoic ring hydroxylation and chiral inversion of enantiomers (Figure 2). Glucuronidation is the dominant mechanism of ketoprofen metabolism, common to all species studied to date, while the importance of hydroxylation and enantiomeric inversion demonstrates a clear species dependence. For example, in rats, enantiomer inversion occurs to a significant extent, accounting for over 75% of the administered dose, and extensive hydroxylation of ketoprofen’s aromatic ring has also been observed in this species [14].

Although glucuronidation is the predominant metabolic pathway for ketoprofen in humans, it is not irreversible. Dexketoprofen is recovered from urine within 12 h, accounting for 73.3–81.9% of the administered dose in unchanged form. This is because ketoprofen glucuronides can be hydrolysed, regenerating the parent compound, so ketoprofen reaches the environment predominantly unchanged.

## 3. Presence of Ketoprofen in Sewage and the Natural Environment

The widespread and long-term use of ketoprofen has led to its ubiquitous presence in the environment, both as the parent compound and as biotransformation products [24,25,26]. At present, ketoprofen is among the most frequently detected pharmaceutical compounds worldwide. Its occurrence has been reported on all continents, including trace concentrations in Antarctic seawater [27].

In aquatic environments, ketoprofen concentrations typically range from 250 ng/L to 6.4 μg/L [1,28]. For example, concentrations recorded in Italian rivers ranged from 170 to 2500 ng/L, whereas in South Africa they varied from 1700 ng/L to 6400 ng/L [1]. In raw wastewater influents, ketoprofen concentrations may reach up to 104 μg/L [29]; however, substantially higher levels, up to 260 μg/L, have been reported in wastewater from India [27].

Ketoprofen is frequently detected in surface and groundwater, hospital and domestic wastewater, and decentralised wastewater treatment systems. Its presence has also been reported in landfill leachate and industrial effluents, largely due to incomplete removal during conventional wastewater treatment processes [2,30]. Ketoprofen has also been identified in drinking water [26]. In addition to aquatic environments, the compound has been detected in soils following irrigation with treated wastewater [2,30].

The enantiomeric distribution of ketoprofen in aquatic environments depends both on the extent of chiral inversion of the R enantiomer during human metabolism and on the preferential biodegradation of the S enantiomer during wastewater treatment. This issue is particularly important given the ecotoxicological relevance of ketoprofen [28].

In surface waters, in addition to ketoprofen itself, transient reactive intermediates and final degradation products are also observed when ketoprofen undergoes UV-induced photodegradation. These products retain the benzophenone chromophore, which may, among other effects, induce photoallergic reactions [26].

Available data indicate that environmental contamination with ketoprofen is global in nature, but its concentrations exhibit marked geographic and regional variations—from surface waters in Europe and Africa to trace amounts recorded even in Antarctic marine waters. This wide distribution demonstrates its high mobility and persistence in the environment, as well as the global nature of pharmaceutical pressure. Significant regional differences are evident primarily in concentration levels, which may result from factors such as drug consumption, over-the-counter availability, population density, degree of urbanization, and, above all, the effectiveness of local wastewater treatment systems. In regions with less effective sanitary infrastructure or a high proportion of decentralized treatment systems, a higher risk of ketoprofen entering surface and groundwater is observed.

In summary, although ketoprofen is a global pollutant, its actual environmental impact is clearly regional. These are determined by differences in drug consumption, wastewater treatment effectiveness, climatic conditions favoring photodegradation, and local biotransformation processes. This diversity highlights the need for regionally tailored environmental risk monitoring and management strategies.

## 4. Ketoprofen Degradation

Conventional wastewater treatment plants are not designed to efficiently remove pharmaceuticals; consequently, most treatment processes are ineffective at removing ketoprofen. For this reason, increasing attention has recently been devoted to optimising degradation pathways of this compound. The main research directions include the development of efficient biodegradation strategies as well as physicochemical methods, particularly those based on advanced oxidation processes (AOPs) [27,31,32,33].

Physicochemical methods are currently regarded as among the most effective approaches for pharmaceutical degradation. Ketoprofen, as a multifunctional compound containing an aromatic ring, is susceptible to ultraviolet radiation. Its degradation under UV irradiation primarily involves direct photolysis and oxidation reactions mediated by hydroxyl radicals. Direct photolysis occurs through the cleavage of the bond between the hydroxyl and carbonyl groups following UV absorption, resulting in the formation of •OH radicals that initiate further advanced oxidation reactions. At higher ketoprofen concentrations, more hydroxyl radicals are generated; therefore, the overall removal rate of ketoprofen from solutions with higher initial concentrations is typically increased [34].

Among the methods with particularly high potential for ketoprofen degradation are advanced oxidation processes such as ozonation, photo-Fenton, photocatalysis, and radiolysis. AOPs are based on the in situ generation of highly reactive, short-lived oxidative species (e.g., H_2_O_2_, •OH, O_2_•^−^, O_3_), which initiate chain oxidation reactions leading to partial degradation or complete mineralisation of organic contaminants [32,33,35].

Illés et al. [36] investigated the degradation of ketoprofen via water radiolysis in solutions saturated with nitrous oxide, induced by γ-radiation emitted from a cobalt-60 source to generate hydroxyl radicals. Reactions between these radicals and ketoprofen yielded compounds with reduced toxicity, as confirmed by toxicity tests using *Daphnia magna* as a model organism. Prolonged irradiation of oxygen-saturated ketoprofen solutions ultimately led to complete mineralisation [36].

Among AOPs, heterogeneous photocatalysis using semiconductor materials activated by high-energy UV radiation is characterised by particularly high efficiency in degrading organic molecules and achieving their final mineralisation to carbon dioxide and water. The photocatalytic properties of semiconductor materials are influenced by various factors, including particle size, band gap width, chemical composition, crystal structure, and surface density of hydroxyl groups [35].

Currently, heterogeneous photocatalytic processes most commonly employ UV radiation and hydroxyl radical generation using titanium dioxide (TiO_2_). Titanium dioxide is one of the most extensively studied photocatalysts due to its high chemical stability, low cost, strong photoactivity, and favourable electron–hole recombination characteristics. When the surface of the solid is irradiated with light possessing energy equal to or greater than its band gap (approximately 3.2 eV), an electron is promoted from the valence band to the conduction band, generating an electron–hole pair. As a result, redox reactions are promoted on the photocatalyst surface, leading to the formation of reactive species such as hydroxyl radicals (•OH), superoxide radicals (O_2_•^−^), and hydroperoxyl radicals (HO_2_•), which are capable of degrading organic compounds and mineralising contaminants to carbon dioxide and water [37].

It has been demonstrated that ketoprofen and its conjugate base are adsorbed onto the TiO_2_ surface due to electrostatic interactions between the negatively charged carboxylate group of ketoprofen molecules and the positively charged surface of titanium dioxide. Subsequently, •OH radicals and other oxidative species generated on the photocatalyst attack the aromatic rings or side chains of ketoprofen, leading to its oxidation [35]. As a non-selective oxidant, •OH tends to react with organic compounds primarily through hydroxyl addition to unsaturated carbon atoms, hydrogen abstraction, and electron transfer reactions [38].

The use of UV radiation at 254 nm for water photolysis in the presence of TiO_2_ as a photocatalyst intensifies oxidation processes, leading to the decarboxylation of ketoprofen into 3-ethylbenzophenone, which is subsequently transformed into other hydroxylated benzophenone derivatives. These compounds may undergo homolytic or heterolytic bond cleavage, forming additional organic transformation products such as 3-(1-carboxyethyl)benzoic acid and various carboxylic acids [39].

**Figure 3:**
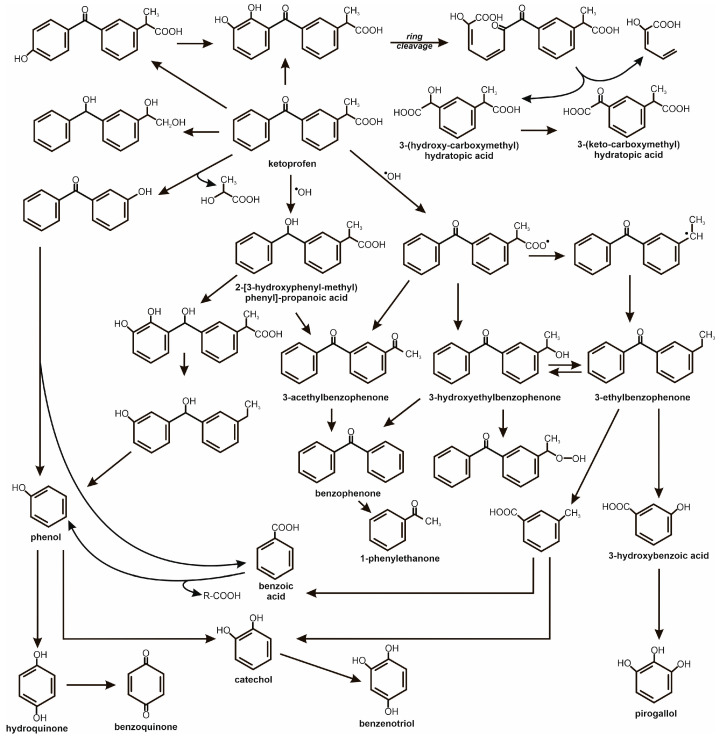
The mechanism of physicochemical degradation of ketoprofen involving free oxygen radicals leading to hydroxylation of aromatic rings and their gradual oxidation to single-ring derivatives [35,38,41,42].

Acosta et al. [35] confirmed that, in UV/TiO_2_ systems, hydroxyl radicals play a dominant role in the photocatalytic degradation and mineralisation of ketoprofen. Its transformation proceeds via a series of hydroxylated intermediates, including hydroxybenzophenone, benzoic acid, hydroquinone, catechol, benzoquinone, benzenetriol, and phenol, following a complex series–parallel reaction pathway. Figure 3 shows the mechanics of the physicochemical degradation of ketoprofen.

The use of persulfates as sources of sulfate radicals, which are highly reactive toward organic compounds, has also resulted in highly efficient ketoprofen degradation, reaching up to 100%. Persulfate activation can be achieved thermally, chemically, or via UV irradiation, with UV activation yielding the most effective transformation of ketoprofen. The observed degradation products primarily include decarboxylated ketoprofen derivatives [32].

Effective degradation of ketoprofen has also been achieved through photo-Fenton and solar photo-Fenton processes. In the conventional Fenton process, hydroxyl radical generation involves the reaction of hydrogen peroxide with iron ions used as catalysts in the form of salts or metal oxides. The application of radiation significantly influences degradation kinetics by photochemically reducing Fe^3+^ to Fe^2+^, thereby restoring the radical generation cycle [40].

Feng et al. [41] generated hydroxyl radicals for the oxidation of ketoprofen using electrochemical methods, including the electro-Fenton reaction and anodic oxidation with platinum (Pt) and boron-doped diamond (BDD) anodes, with a carbon felt cathode. The most effective degradation, leading to complete mineralisation, was achieved via anodic oxidation using a boron-doped diamond anode. In anodic oxidation, heterogeneous hydroxyl radicals (•OH) are produced by electrochemical discharge of water or hydroxide ions at anodes with a high oxygen evolution overpotential. In the case of BDD anodes, •OH radicals are physically adsorbed and therefore more readily available than on Pt anodes, where they are chemically adsorbed, which is reflected in the higher efficiency of the process. Analysis of intermediate products enabled the proposal of degradation pathways involving aromatic intermediates, including 3-hydroxybenzoic acid, pyrogallol, catechol, benzophenone, benzoic acid, and hydroquinone. Although this initially increased solution toxicity, further degradation led to the formation of aliphatic acids—formic, acetic, oxalic, glycolic, and glyoxylic acids—ultimately reducing toxicity [14].

Ultrasonic treatment has also long been regarded as a promising AOP for removing pharmaceutical compounds from water and is considered a green technology because it does not use chemical additives. Ultrasound induces the formation of cavitation bubbles in water, which undergo transient collapse events. Three reaction zones are generally recognised: the hot spot within the bubble, the bubble–water interface, and the bulk solution. During cavitation collapse, extreme temperatures (>5000 K) and pressures (>500 atm) can be achieved, leading to the pyrolysis of water and the formation of reactive oxygen species, such as •OH, •O, and H•. Reactive radicals generated in the hot spot and at the bubble–water interface can diffuse into the bulk solution. Nonvolatile and hydrophilic compounds are mainly degraded in the bulk solution via radical oxidation, whereas hydrophobic compounds such as ketoprofen more readily diffuse to the bubble–water interface or even into the vapour phase, where they undergo pyrolysis or radical oxidation. Consequently, the ultrasonic degradation mechanism depends strongly on compound properties, such as pKa, octanol–water partition coefficient, and Henry’s law constant [38,43].

Gao et al. [38] observed rapid degradation of ketoprofen in water under ultrasonic irradiation at 555 kHz. The degradation mechanism was dominated by hydroxyl radical oxidation at the bubble–water interface. Analysis of the resulting products revealed three major degradation pathways: (i) hydroxylation initiated by •OH attack on aromatic ring carbon atoms, leading to monohydroxylated products and subsequent meta-ring cleavage; (ii) decarboxylation, where hydrogen abstraction from carboxyl groups produces ketoprofen radicals followed by CO_2_ elimination and benzyl radical formation, ultimately yielding 3-ethylbenzophenone and its transformation products; and (iii) decarboxylation followed by molecular rearrangement and hydroxylation [38].

Despite the high efficiency of physicochemical methods in ketoprofen degradation, their major drawbacks include high operational costs and the potential formation of toxic by-products. Consequently, increasing research efforts are directed toward identifying microorganisms capable of degrading ketoprofen and enhancing the efficiency of bioprocesses as more sustainable alternatives to physicochemical treatment methods.

**Figure 4:**
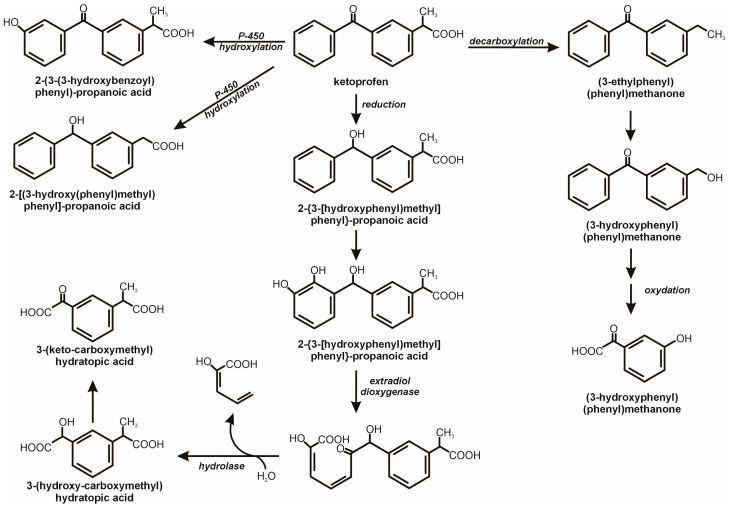
Biological degradation of ketoprofen involving monooxygenases and cleavage dioxygenases leading to single-ring derivatives [44,45,46].

One of the first comprehensive studies documenting the biodegradation of ketoprofen was conducted by Quintana et al. [44]. The authors demonstrated the complete (100%) transformation of this drug in a mixed activated sludge culture. Analysis of the intermediate products produced indicated that the degradation process involved 2-[3-[carboxy(hydroxy)methyl]phenyl]propanoic acid and 2-(3-oxalophenyl)propanoic acid. Based on the obtained results, a ketoprofen degradation pathway was proposed, in which the initiating step is dioxygenase-catalysed cleavage of the benzyl ring, followed by hydrolysis of the resulting side chain (Figure 4) [44].

Ismail et al. [45] demonstrated that ketoprofen biodegradation by a mixed consortium of microorganisms is more efficient in the absence of light than under irradiation. This phenomenon was attributed to the phototoxicity of ketoprofen and its conversion to more toxic photolysis products. The microorganisms responsible for the degradation were identified as *Raoultella ornithinolytica* B6, *Pseudomonas aeruginosa* JPP, and *Pseudomonas* sp. P16, and *Stenotrophomonas* sp. 5LF 19TDLC. Among the degradation products, (3-ethylphenyl)(phenyl)methanone, (3-hydroxyphenyl)(phenyl)methanone, and (3-hydroxyphenyl)(oxo)acetic acid were detected. Toxicity tests using algae as model organisms confirmed a significant reduction in metabolite toxicity compared to the parent compound, resulting in an 82% reduction in mortality [45].

It has also been demonstrated that ketoprofen biodegradation can be enhanced by a microbiologically induced, nonenzymatic Fenton-type reaction. A marine microbial consortium dominated by *Halomonas*, *Marinobacter*, *Owenweeksia*, and *Oceanimonas* degraded ketoprofen more effectively in the presence of casein acids than under glucose-based culture conditions. The presence of casein acids promoted the generation of hydrogen peroxide (H_2_O_2_) and hydroxyl radicals (•OH). The microorganisms in the consortium exhibited amino acid oxidase activity, which likely participated in this process [42].

Meanwhile, the *Halomonas* and *Marinobacter* strains were characterised by their ability to reduce Fe(III) to Fe(II). These results indicate a reaction analogous to the Fenton reaction, in which the Fe(III)/Fe(II) cycle promoted increased •OH radical production and intensified ketoprofen degradation. This reaction was mainly involved in the initial stage of ketoprofen hydroxylation, leading to the formation of 2-(3-(2-hydroxybenzoyl)phenyl)propanoic acid or its isomer, 2-(3-(3-hydroxybenzoyl)phenyl)propanoic acid, and 2-(3-(2-hydroxy-3-methylbenzoyl)phenyl)propanoic acid. The latter compound then underwent meta-cleavage and hydrolysis to 2-hydroxymuconic semialdehyde and 3-(ketocarboxymethyl)hydratopic acid, which was then reduced to 3-(hydroxycarboxymethyl)hydratopic acid. These products were not detected in the simulated Fenton reaction, and their formation was attributed to enzymatically catalyzed reactions involving the studied consortium [42]. A similar degradation pathway was observed during the degradation of ketoprofen by a *Bacillus paralicheniformis* strain isolated from a soda lake sediment, with the cytochrome P-450 enzymatic system responsible for the initial hydroxylation step [46].

The involvement of white rot fungi, such as *Trametes versicolor*, in the degradation of ketoprofen is also noteworthy. A dose of 11 mg/L of this drug was shown to be transformed by the fungi within 24 h, with the main metabolite observed after this time being 2-[(3-hydroxy(phenyl)methyl)phenyl]propanoic acid. After seven days of incubation, a significant decrease in the concentration of this product was observed, suggesting further mineralisation of the compound [47].

In recent years, enzymatic biocatalysis has gained significant importance in the bioremediation of resistant pharmaceuticals, including ketoprofen. The most commonly used enzymes are laccases from *Trametes versicolor*, which catalyse the radical oxidation of aromatic hydrogen donors in the presence of molecular oxygen [48,49]. The immobilisation of laccases on natural, low-cost carriers is gaining increasing interest, enabling the simultaneous adsorption and biodegradation of contaminants. For example, using date pits (PDS) as a laccase carrier maintained enzyme stability over multiple reaction cycles and removed 25 mg/L of ketoprofen within 4 h. The observed reduction in drug concentration resulted from a synergistic interaction between adsorption and enzymatic degradation [49].

In summary, the analysis of ketoprofen degradation indicates significant limitations in the use of both physicochemical and biological methods for its removal. The most significant barriers associated with physicochemical methods include high costs, the nonspecificity of the generated radicals, and the formation of difficult-to-predict intermediates of uncertain toxicity. Furthermore, these techniques are difficult to implement in real-world conditions, and photodegradation without appropriate catalysts is inherently inefficient. Furthermore, ketoprofen biodegradation is slow, and the number of microorganisms capable of degrading it effectively is limited. Moreover, biological degradation is primarily carried out in laboratory systems under conditions significantly different from those in the environment, making it impossible to predict its actual efficiency under environmental conditions. Laboratory strains are often sensitive to changing environmental conditions and competition from indigenous microbiota. Consequently, these limitations currently prevent the effective implementation of these methods for removing ketoprofen from the environment, despite their perceived potential.

For biological methods to fully manifest their application potential, it is essential to conduct research in systems that faithfully replicate natural treatment processes—particularly in reactor systems with mixed microbial populations and under variable environmental conditions. At the same time, continuous analysis of degradation intermediates and their toxicity assessment is essential and forms the basis for a reliable environmental risk assessment. Modern wastewater treatment plants should consider integrating physicochemical and biological methods, which involve initiating the degradation of compounds using advanced oxidation processes and then further removing them through microbiological processes. However, the risk associated with the toxicity of intermediates formed during the physicochemical stages must be considered. Therefore, monitoring the toxicity of these compounds and appropriately targeting the process by selecting appropriate sorbents and catalysts is crucial.

## 5. Toxicity of Ketoprofen to Non-Target Organisms

Ketoprofen, like many other pharmaceutical compounds, can affect living organisms even at low concentrations. Once released into the natural environment, it may exert adverse effects on organisms that are not the intended targets of its therapeutic action. It has been demonstrated that ketoprofen can cross biological membranes, accumulate in organisms, and induce ecotoxicological effects. To assess the environmental risk posed by this compound, numerous studies have evaluated its acute toxicity and the consequences of chronic exposure in living organisms [30].

The toxicity of chemical substances is commonly classified according to the criteria established by the Globally Harmonised System of Classification and Labelling of Chemicals (GHS), which is used to assess hazards to the aquatic environment. Within this system, four hazard categories are defined based on L(E)C_50_ (Lethal (Effect) Concentration 50%) values, which reflect the degree of toxicity of a given substance [27,50,51]. Acute toxicity describes the hazard posed by a substance following a single, short-term exposure. However, in ecotoxicological assessments, evaluating chronic toxicity is of primary importance, as it addresses the long-term effects of continuous exposure to low concentrations of a given compound. In contrast to acute toxicity, chronic toxicity assessments focus mainly on sublethal effects rather than mortality [27]. Observed outcomes of chronic exposure may include oxidative stress, disruption of endocrine and reproductive systems, impaired organ function, and tissue damage. Additionally, disturbances in metabolic processes, alterations in immune responses, and genotoxic effects have also been reported [52]. In the case of ketoprofen, adverse effects have been demonstrated across a wide range of organisms, including invertebrates, vertebrates, plants, and various groups of microorganisms [53,54,55].

### 5.1. The Negative Impact of Ketoprofen on Microorganisms

Prokaryotic organisms play a crucial role in environmental functioning by participating in biogeochemical cycles and contributing to the detoxification of pollutants. Therefore, the condition of the microflora inhabiting a given biocenosis is fundamental to ecosystem stability. Analyses of the effects of ketoprofen on soil microbial activity—assessed through glucose-induced respiration, the abundance of culturable bacteria and fungi, and the activity of enzymes such as dehydrogenases, acid and alkaline phosphatases, and urease—have shown that, in most cases, ketoprofen at a concentration of 10 mg/kg of soil caused an initial inhibition of microbial respiration, enzymatic activity, and microbial abundance. Subsequently, stimulation of these processes was observed most often after 30 days of incubation. This effect may result from the initial elimination of ketoprofen-sensitive microbial populations followed by their replacement with resistant strains [56]. Studies by Pawłowska et al. [57] investigating the impact of ketoprofen on soil bacteria and fungi demonstrated that its presence increased the activity of antioxidant enzymes, including catalase, superoxide dismutase, and guaiacol peroxidase. This indicates the induction of oxidative stress in soil environments exposed to ketoprofen.

The sensitivity of *Escherichia coli* to ketoprofen, manifested by the induction of oxidative stress, was exploited by Matejczyk et al. [58] to construct a biosensor based on green fluorescent protein (GFP) fused with promoters of the *recA*, *katG*, and *sodA* genes. This system enabled monitoring of the cytotoxic and genotoxic effects of ketoprofen. The *SOS*, *SoxRS*, and *OxyR* regulons, controlling the expression of *recA*, *sodA*, and *katG*, were induced in response to reactive oxygen species generated in the presence of ketoprofen. These findings confirmed that ketoprofen, similarly to other non-steroidal anti-inflammatory drugs, can induce oxidative stress in bacteria [58,59].

It has also been demonstrated that ketoprofen adversely affects cell membrane integrity. This leads to the irreversible inhibition of nitrite production by the ammonia-oxidising bacterium *Nitrosomonas europaea*, thereby negatively impacting the nitrogen cycle in natural ecosystems [60].

Data from biotests indicated that *Vibrio fischeri* was more sensitive to dexketoprofen than to racemic ketoprofen, suggesting enantioselective toxicity partially attributable to the S(+) enantiomer present in the racemate. Exposure to the S(+) enantiomer of ketoprofen resulted in inhibition of bioluminescence with an EC_50_ value of 1046 mg/L [30]. In contrast, complete inhibition of luminescence in *Aliivibrio fischeri* was observed at 40 mg/L, and the inhibition curve for ketoprofen showed a more pronounced toxic effect than those of other NSAIDs, such as naproxen or diclofenac [61]. Moreover, a hormetic effect was observed following exposure to racemic ketoprofen [30].

Tyumina et al. [27] demonstrated that, in the ketoprofen-degrading strain *Rhodococcus erythropolis*, prolonged exposure to ketoprofen can induce morphological alterations, such as changes in cell shape or size. These adaptations may mitigate the negative effects of ketoprofen on bacterial cells’ metabolic state.

An important aspect of ketoprofen’s impact on bacterial communities is its interference with quorum sensing. Ketoprofen has been shown to disrupt quorum sensing in *Chromobacterium violaceum* and *Pseudomonas aeruginosa*. In *C. violaceum*, this disruption is characterised by inhibition of quorum–sensing–regulated processes, including biofilm formation, cell aggregation, and violacein production. In the pathogenic bacterium *P. aeruginosa*, quorum-sensing interference reduces the production of virulence factors and impairs biofilm formation [55,62,63].

### 5.2. Ketoprofen Toxicity to Invertebrates

The presence of ketoprofen in aquatic environments may significantly disrupt the functioning of numerous invertebrate organisms inhabiting these ecosystems. To date, ecotoxicological studies on invertebrates have primarily focused on classical toxicity endpoints, such as mortality or immobilisation, and selected biochemical parameters [64]. The most extensive body of data originates from studies conducted on the model organism *Daphnia magna*, as well as on other crustaceans and molluscs [30,64,65].

Studies on *Daphnia magna* demonstrated that its response to ketoprofen depends on both concentration and exposure duration. LC_50_ values for ketoprofen ranged from 11.02 to 100 mg/L, depending on the exposure duration. Ketoprofen exposure induced numerous physiological and behavioural alterations in daphnids, including a reduction in heart rate, disturbances in reproduction, feeding, and locomotion, as well as changes in biochemical parameters [30,64,65]. The inhibition of swimming speed observed even at low ketoprofen concentrations may result from adverse effects on the motor neurons innervating the second antennae, which are responsible for locomotion. Exposure also altered jumping frequency, with a transient increase at the lowest concentration—indicative of a hormetic response—followed by inhibition at higher levels, likely due to disrupted neuromuscular function. Additionally, abnormal swimming patterns characterised by alternating forward and backward movements suggested impairments in spatial orientation and environmental perception, which under natural conditions may compromise migration, predator avoidance, and foraging efficiency [64]. However, Alkimin et al. [65] did not observe any effects of low ketoprofen concentrations on the feeding ability of *Daphnia magna*.

Ketoprofen also affects a range of biochemical parameters, including Na^+^/K^+^-ATPase activity and components of the antioxidant defence system, such as superoxide dismutase, catalase, glutathione, and glutathione S-transferase, as well as cyclooxygenase activity [64,65]. These disturbances may reduce heart rate in daphnids, likely due to impaired neuronal transmission or oxidative damage to cardiac neurons, thereby affecting hemolymph circulation. A decrease in thoracic limb activity was also observed, which may result from reduced contractile strength of thoracic muscles. As these appendages play a crucial role in food filtration and ventilation, their dysfunction may cause feeding disturbances and hypoxia. The mechanism underlying the inhibition of thoracic limb activity appears to be similar to that responsible for swimming impairments [64].

Nevertheless, Alkimin et al. [65] demonstrated that, at environmentally relevant concentrations, the toxic effects of ketoprofen were mainly limited to biochemical alterations, without clear extrapolation to physiological or population-level effects. Despite no changes in life-history traits or feeding behaviour, ketoprofen activated the antioxidant system of *D. magna*, increasing catalase activity, interpreted as a response to elevated reactive oxygen species levels. At the same time, induction of the biotransformation system was observed, manifested by increased glutathione S-transferase activity, suggesting that ketoprofen may be excreted after conjugation with glutathione. This effect was not observed at the lowest concentrations, indicating the occurrence of hormesis—at low exposure levels, hydroxylation processes may have been sufficient to detoxify the pharmaceutical, rendering phase II glutathione conjugation unnecessary [65].

It was also demonstrated that ketoprofen reduced the fertility of *Daphnia dubia*, with the observed toxic effect being enantioselective. Racemic ketoprofen at a concentration of 1 mg/L caused a 24.9% reduction in fertility, whereas (S)-ketoprofen at a concentration of 100 µg/L resulted in a 28.8% decrease [30].

Due to the presence of a conserved cyclooxygenase synthase in crustaceans, exposure to ketoprofen led to a pronounced, acute inhibition of this enzyme, even at low concentrations, which may be of key importance for reproductive impairment by interfering with the eicosanoid biosynthesis pathway [65].

It should be emphasised that the ketoprofen concentrations used in the cited studies were comparable to those detected in aquatic environments, suggesting that the observed behavioural, biochemical, and physiological disturbances may occur under natural exposure conditions [64]. Considering the crucial role of daphnids in freshwater ecosystems—where the genus *Daphnia* is regarded as a dominant primary consumer within the zooplankton community—even subtle impairments in their functioning may disrupt trophic interactions [64,65].

High sensitivity to ketoprofen was also reported in another small crustacean, *Heterocypris incongruens*. Pawłowska et al. [57] observed significant reductions in growth and survival in this species, which is considered a bioindicator organism due to its high sensitivity. This vulnerability is associated with its feeding behaviour at the sediment–water interface, which exposes it to both dissolved and particulate contaminants that may enter the organism via the diet.

Similarly, the mussel *Mytilus galloprovincialis* has been shown to be sensitive to ketoprofen at environmentally relevant concentrations. Analyses of molecular, biochemical, and cellular biomarkers revealed alterations in immunological parameters, genotoxic effects, modulation of lipid metabolism, and changes in cellular turnover. Among the wide range of biological responses examined, dose-dependent destabilisation of lysosomal membrane integrity emerged as a particularly sensitive biomarker for detecting adverse effects of ketoprofen. In addition, modulation of the immune system was confirmed, evidenced by a significant decrease in the granulocyte-to-hyalinocyte ratio and a concurrent inhibition of phagocytic capacity. These effects were likely associated with the lower phagocytic ability of hyalinocytes compared to granulocytes, which normally constitute the dominant hemocyte type in *M. galloprovincialis* hemolymph and are responsible for cellular immunity via phagocytosis and cytotoxic reactions. Furthermore, slight changes in acyl-CoA oxidase activity were observed, potentially altering energy metabolism. Genotoxic damage was also detected, as evidenced by increased DNA fragmentation and a higher frequency of micronuclei following ketoprofen exposure [66].

Moreover, ketoprofen has been shown to inhibit behavioural fever—an adaptive response observed in some ectothermic animals that involves selecting warmer environments to increase body temperature in response to bacterial infection or the presence of pyrogenic substances such as bacterial lipopolysaccharide. This phenomenon was observed in freshwater snails *Planorbarius corneus*, in which the behavioural fever response was completely suppressed in the presence of ketoprofen [67].

Given the widespread occurrence of ketoprofen in aquatic environments at environmentally realistic concentrations, comprehensive information on its effects on crustaceans and molluscs is essential for an accurate assessment of the ecotoxicological risk posed by this pharmaceutical compound.

### 5.3. Ketoprofen Toxicity to Non-Target Vertebrates

Following the mass extinction of South Asian vultures caused by diclofenac, the safety of ketoprofen was evaluated in vultures. Toxicity tests conducted on *Gyps coprotheres* and *G. africanus* revealed that ketoprofen induced mortality and severe renal toxicity at doses of 1.5–5 mg/kg. Exposed birds exhibited elevated plasma uric acid, alanine aminotransferase, and sodium levels, extensive visceral gout, and pronounced kidney damage associated with urate crystal deposition. Mortality rates reached 7 of 11 individuals at 5 mg/kg, indicating that ketoprofen is lethal at doses likely encountered in the wild through consumption of contaminated livestock carcasses. Residue analyses from carcass dumps in India confirmed the presence of ketoprofen at environmentally relevant concentrations, with substantially higher accumulation in kidney tissue. These findings raise serious concerns regarding the veterinary use of ketoprofen and other NSAIDs, highlighting the need for strict regulation to prevent further declines in vulture populations [53].

Most studies investigating the toxicity of ketoprofen to vertebrates have been conducted on various fish species, with bioaccumulation being the most frequently reported phenomenon. Ketoprofen has been detected, among others, in the cardiac muscle of the riverine fish *Liza ramada*, in the tissues of marine fish such as *Mugil curema* and *Centropomus undecimalis* at concentrations up to 12 ng/kg, as well as in commonly consumed species, including Atlantic salmon (*Salmo salar*) and rainbow trout (*Oncorhynchus mykiss*), where substantially higher levels ranging from 80 to 120 µg/kg were reported [67,68,69,70]. In contrast, Mennillo et al. [28] demonstrated that neither racemic ketoprofen nor dexketoprofen accumulated in the muscle tissue of Atlantic salmon.

Studies using the model organism *Danio rerio* indicate that ketoprofen can induce a variety of toxic effects in aquatic fauna. Numerous developmental abnormalities have been reported, including cardiac oedema, delayed hatching, and scoliosis, at concentrations ranging from 1 to 100 mg/L. At higher concentrations (10–100 mg/L), additional effects were observed, such as cardiac elongation, a significant reduction in heart rate, increased embryonic mortality, and yolk sac oedema. Moreover, a marked decrease in hepatic Na^+^/K^+^-ATPase activity was observed across all tested concentrations. Reduced activity of this enzyme, which is essential for neuronal transmission, may lead to a decreased heart rate in zebrafish [64,71].

Investigations encompassing embryonic and juvenile stages of *Danio rerio* revealed a markedly higher sensitivity of embryos to ketoprofen compared with juvenile forms. The LC_50_ value after 96 h of exposure for juveniles was 632.3 mg/L, whereas after 144 h of exposure, it was only 6.44 mg/L for embryos. This differential sensitivity among developmental stages may result from the immaturity of enzymatic systems in embryos, differences in uptake pathways, metabolic variability, and the presence of the chorion, which may limit ketoprofen penetration into target organs. Embryotoxicity assays demonstrated growth inhibition after 144 h of exposure in all experimental groups, as well as oedema at concentrations exceeding 6 mg/L and embryonic immobilisation at concentrations ≥9 mg/L [72].

Mennillo et al. [30] demonstrated that, in studies conducted on the hepatocellular carcinoma cell line derived from the liver of *Poeciliopsis lucida* (PLHC-1), the toxic effects of ketoprofen were enantioselective. Although neither racemic ketoprofen nor dexketoprofen exhibited cytotoxic effects on PLHC-1 cells, compound-, time-, and concentration-dependent differences were observed in cytochrome P450 1A (CYP1A) and glutathione S-transferase activity. The effects of racemic ketoprofen and dexketoprofen on CYP1A differed at both transcriptional and catalytic levels, suggesting distinct intracellular mechanisms of action. During phase II biotransformation, a significant increase or decrease in glutathione S-transferase activity was observed in cells exposed to racemic ketoprofen or dexketoprofen, respectively. Clear differences were also noted in their interaction with efflux pumps: racemic ketoprofen increased efflux pump expression, whereas dexketoprofen reduced it, except at the highest tested concentration. In contrast, toxicological studies examining biotransformation processes and oxidative stress responses in the gills and liver of Atlantic salmon did not detect significant differences between racemic ketoprofen and its enantiomer, dexketoprofen, revealing organ-specific responses. In the gills, a pronounced inhibitory effect was observed at both transcriptional and enzymatic levels on biotransformation and oxidative stress responses. In the liver, biotransformation responses were significantly enhanced at the transcriptional and translational levels, while the associated enzymatic activity was concurrently suppressed [28].

It should be emphasised that, under natural environmental conditions, the overall toxicity of ketoprofen may be modulated by processes occurring in aquatic systems, such as photolysis—particularly in the presence of bicarbonates—which can lead to the formation of more toxic transformation products. Furthermore, biological responses may be more complex due to unpredictable additive and synergistic interactions with other chemical contaminants commonly present in aquatic environments. A notable example is provided by studies on the combined effects of cadmium and ketoprofen at environmentally relevant concentrations to sexually mature *Danio rerio*. These studies demonstrated that combined exposure of parental fish to Cd and ketoprofen increased chemical accumulation rates, intensified gonadal tissue damage, and significantly reduced reproductive capacity. Moreover, these adverse effects were transmitted to the F1 generation, as evidenced by reduced hatching success, body deformities, and altered transcription of genes associated with the thyroid axis [73].

### 5.4. Impact of Ketoprofen on Plants

To date, relatively little is known about the negative effects of ketoprofen on plants. However, the available literature indicates that this compound may affect not only aquatic flora but also terrestrial plants growing in soil contaminated with ketoprofen, for example, as a result of fertilisation with sewage sludge or domestic wastewater [54,57,74].

The negative effects of ketoprofen have been observed, among others, in the green alga *Pseudokirchneriella subcapitata* [54]. Furthermore, Mennillo et al. [30] demonstrated that the EC_50_ value for algae was significantly higher for racemic ketoprofen (0.24 mg/L) than for dexketoprofen (0.066 mg/L), classifying the latter in hazard category I.

Studies by Wang et al. [74] confirmed earlier observations obtained in animal studies, indicating that low concentrations of ketoprofen may induce a hormetic effect. Ketoprofen at 0.5 mg/L slightly stimulated rice seedling growth (*Oryza sativa*), whereas higher concentrations significantly inhibited growth by reducing biomass and causing root damage. A concentration of 20 mg/L resulted in a decrease in photosynthetic pigment content (chlorophyll a and b as well as carotenoids), substantial damage to cell membranes, and alterations in the expression of genes involved in chlorophyll biosynthesis. In addition, increased levels of reactive oxygen species, malondialdehyde, and proline were observed, indicating the induction of oxidative stress in chloroplasts, mitochondria, and cell membranes. Similar effects of ketoprofen were also observed in barley (*Hordeum vulgare*) [57,73]. In contrast, in *Lemna minor*, activation of oxidative stress response mechanisms was observed in the presence of ketoprofen, manifested by increased activities of catalase, glutathione S-transferases, and carbonic anhydrase. However, no significant effect of ketoprofen on chlorophyll and carotenoid levels was observed in this plant, which supports earlier findings that plant responses to ketoprofen depend not only on concentration and exposure time but also strongly on species [65].

In summary, analysis of available data on ketoprofen toxicity to non-target organisms reveals significant research and methodological gaps. The problem lies in the lack of standardized toxicity testing and the predominance of studies using high, unrealistic ketoprofen concentrations and short incubation times, which only determine acute toxicity. Sublethal effects remain significantly underestimated due to the lack of studies on long-term exposure of organisms to low environmental concentrations of ketoprofen. A significant shortcoming of the conducted studies is the failure to consider emerging intermediates of biological and photochemical degradation and possible synergistic reactions between ketoprofen and its metabolites and other environmental contaminants in the ecological risk assessment. Furthermore, a significant shortcoming of the conducted research is the lack of multi-species studies, resulting in limited information on ketoprofen toxicity at the ecosystem level. A summary of key knowledge gaps is presented in Table 1.

It is also important to emphasize the complete lack of explanation in the literature for an interesting effect confirmed in studies on ketoprofen toxicity—the phenomenon of hormesis observed at low concentrations of this substance. This phenomenon requires further in-depth research, as very low concentrations of ketoprofen are most common in the natural environment, and it is under such conditions that the hormetic effect can occur. A complete explanation of this mechanism could have significant practical implications, including reducing the need for costly wastewater treatment to remove micropollutants.

Table 2 compares environmental concentrations of ketoprofen with the described toxic effects.

**Figure 5:**
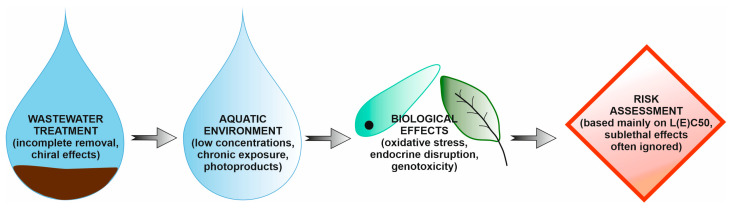
Limitations of standard ecotoxicological risk assessment for ketoprofen.

Most of the analyzed studies are based on in vitro studies, which severely limit the extrapolation of results to natural conditions. Limitations in determining the ecotoxicological risk of ketoprofen are shown in Figure 5.

Furthermore, the lack of correlation between the risk of ketoprofen photolysis products is a significant problem. It should be emphasized that results based on acute toxicity tests cannot be used to determine the absence of ecological risk.

## 6. Conclusions

Ketoprofen, a known cyclooxygenase inhibitor commonly used in pain management, enters the environment and undergoes biological and photochemical transformation. It can also negatively impact organisms inhabiting it, from microorganisms to vertebrates and vascular plants. Low concentrations of ketoprofen can induce hormesis in both animals and plants. High concentrations can cause cellular damage, often resulting from oxidative stress. Therefore, it is crucial to monitor ketoprofen concentrations in the environment and its impact on non-target organisms, and to seek effective methods for its degradation. It should be emphasized that, despite bacteria’s potential to degrade ketoprofen, knowledge of the microbiological mechanisms underlying its degradation remains fragmented. Further research is needed to integrate the analysis of chemical transformations with the assessment of their toxicological effects. Although the ability to transform ketoprofen has been reported in bacteria and fungi, the lack of data on the fate of the resulting products and their toxicity prevents a clear assessment of the process’s safety.

## Figures and Tables

**Table 1:** Summary of available ecotoxicological data for ketoprofen and key knowledge gaps.

Level of Biological Organization	Type of Study	Typical Endpoint	Concentration Range	Key Limitation/Critical Remarks
Microorganisms	acute/chronic	enzymatic activity, growth and respiratory inhibition	ng/L–mg/L	short exposure periods, limited environmental realism
Invertebrates	acute/chronic	mortality, reproduction, immobilization, biochemical parameters	µg/L–mg/L	sublethal effects often overlooked
Vertebrates	acute/chronic	physiological and endocrine disruption, oxidative stress, mortality	µg/L–mg/L	focus on lethality, laboratory models, limited ecological relevance
Plants	chronic	growth, photosynthesis	µg/L–mg/L	metabolites and photoproducts rarely considered
Multispecies systems	-	-	-	sever lack of data; ecosystem level effects unknown

**Table 2:** Comparison of environmental concentrations of ketoprofen with reported toxicological thresholds.

Context	Concentration Range	Effect Type	Organism	Conclusion
aquatic environment	ng/L–µg/L	-	-	real exposure
acute tests	>mg/L	mortality	microorganisms, *Daphnia*, fishes	low sensitivity
chronic studies	µg/L	sublethal	invertebrates	effects at environmental concentrations
photolysis products	low µg/L	photoallergic	-	underestimated risk

## Data Availability

No new data were created or analyzed in this study.

## References

[1] Wang J., Zhao S.Q., Zhang M.Y., He B.S. (2018). Targeted eco-pharmacovigilance for ketoprofen in the environment: Need, strategy and challenge. Chemosphere.

[2] Xu J., Chen W., Wu L., Chang A.C. (2009). Adsorption and degradation of ketoprofen in soils. J. Environ. Qual..

[3] Mohammadpour R., Dobrovolskaia M.A., Cheney D.L., Greish K.F., Ghandehari H. (2019). Subchronic and chronic toxicity evaluation of inorganic nanoparticles for delivery applications. Adv. Drug Deliv. Rev..

[4] Lagunin A., Zakharov A., Filimonov D., Poroikov V. (2011). QSAR modelling of rat acute toxicity on the basis of PASS prediction. Mol. Inform..

[5] Okomoda V., Solomon S.G., Ataguba G.A., Ayuba V.O., Asuwaju F.P. Acute toxicity test in aquaculture: A review. Proceedings of the 28th Annual Conference of the Fisheries Society of Nigeria (FISON).

[6] Erhirhie E.O., Ihekwereme C.P., Ilodigwe E.E. (2018). Advances in acute toxicity testing: Strengths, weaknesses and regulatory acceptance. Interdiscip. Toxicol..

[7] Damalas C.A., Koutroubas S.D. (2016). Farmers’ exposure to pesticides: Toxicity types and ways of prevention. Toxics.

[8] Isnard P., Flammarion P., Roman G., Babut M., Bastien P., Bintein S., Essermeant L., Ferard J.F., Gallotti-Schmitt S., Saouter E. (2001). Statistical analysis of regulatory ecotoxicity tests. Chemosphere.

[9] Gupta J., Kumari R., Hansraj (2023). A review on new uses of ketoprofen and its role in clinical practices. Int. J. Res. Eng. Sci. (IJRES).

[10] Ong A.L., Kamaruddin A.H., Bhatia S. (2005). Current technologies for the production of (S)-ketoprofen: Process perspective. Process Biochem..

[11] Rajamohan R., Kamaraj E., Muthuraja P., Murugavel K., Govindasamy C., Prabakaran D.S., Malik T., Lee Y.R. (2024). Enhancing ketoprofen’s solubility and anti-inflammatory efficacy with safe methyl-β-cyclodextrin complexation. Sci. Rep..

[12] Sheng J.J., Kasim N.A., Chandrasekharan R., Amidon G.L. (2006). Solubilization and dissolution of insoluble weak acid, ketoprofen: Effects of pH combined with surfactant. Eur. J. Pharm. Sci..

[13] Soto R., Svärd M., Verma V., Padrela L., Ryan K., Rasmuson Å.C. (2020). Solubility and thermodynamic analysis of ketoprofen in organic solvents. Int. J. Pharm..

[14] Barbanoj M.J., Antonijoan R.M., Gich I. (2001). Clinical pharmacokinetics of dexketoprofen. Clin. Pharmacokinet..

[15] Levoin N., Blondeau C., Guillaume C., Grandcolas L., Chretien F., Jouzeau J.Y., Benoit E., Chapleur Y., Netter P., Lapicque F. (2004). Elucidation of the mechanism of inhibition of cyclooxygenases by acyl-coenzyme A and acylglucuronic conjugates of ketoprofen. Biochem. Pharmacol..

[16] Blobaum A.L., Marnett L.J. (2007). Structural and functional basis of cyclooxygenase inhibition. J. Med. Chem..

[17] Rouzer C.A., Marnett L.J. (2020). Structural and chemical biology of the interaction of cyclooxygenase with substrates and non-steroidal anti-inflammatory drugs. Chem. Rev..

[18] Kozlowska M., Rodziewicz P., Kaczmarek-Kedziera A. (2017). Structural stability of diclofenac vs. inhibition activity from ab initio molecular dynamics simulations. Comparative study with ibuprofen and ketoprofen. Struct. Chem..

[19] Selinsky B.S., Gupta K., Sharkey C.T., Loll P.J. (2001). Structural analysis of NSAID binding by prostaglandin H_2_ synthase: Time-dependent and time-independent inhibitors elicit identical enzyme conformations. Biochemistry.

[20] Palomer A., Pascual J., Cabré M., Borràs L., González G., Aparici M., Carabaza A., Cabré F., García M.L., Mauleón D. (2002). Structure-based design of cyclooxygenase-2 selectivity into ketoprofen. Bioorganic Med. Chem. Lett..

[21] Marseglia G.L., Ciprandi G. (2023). Clinical use of ketoprofen lysine salt: A reappraisal in adolescents with acute respiratory infections. Allergol. Immunopathol..

[22] Kantor T.G. (1986). Ketoprofen: A review of its pharmacologic and clinical properties. Pharmacother. J. Hum. Pharmacol. Drug Ther..

[23] Rencber S., Karavana S., Ozyazici M. (2009). Bioavailability file: Ketoprofen. FABAD J. Pharm. Sci..

[24] Brune K., Patrignani P. (2015). New insights into the use of currently available non-steroidal anti-inflammatory drugs. J. Pain Res..

[25] Izadi P., Izadi P., Salem R., Papry S.A., Magdouli S., Pulicharla R., Brar S.K. (2020). Non-steroidal anti-inflammatory drugs in the environment: Where were we and how far we have come?. Environ. Pollut..

[26] Altharawi A., Abdel-Gawad S.A. (2025). Environmental sustainability study for the determination of ketoprofen in the presence of its main photo-degradation products in river water using solid-contact electrodes. Chemosensors.

[27] Tyumina E., Subbotina M., Polygalov M., Tyan S., Ivshina I. (2023). Ketoprofen as an emerging contaminant: Occurrence, ecotoxicity and (bio)removal. Front. Microbiol..

[28] Mennillo E., Pretti C., Cappelli F., Luci G., Intorre L., Meucci V., Arukwe A. (2020). Novel organ-specific effects of ketoprofen and its enantiomer, dexketoprofen on toxicological response transcripts and their functional products in salmon. Aquat. Toxicol..

[29] Almeida B., Oehmen A., Marques R., Brito D., Carvalho G., Crespo M.T.B. (2013). Modelling the biodegradation of non-steroidal anti-inflammatory drugs (NSAIDs) by activated sludge and a pure culture. Bioresour. Technol..

[30] Mennillo E., Arukwe A., Monni G., Meucci V., Intorre L., Pretti C. (2018). Ecotoxicological properties of ketoprofen and the S(+)-enantiomer (dexketoprofen): Bioassays in freshwater model species and biomarkers in fish PLHC-1 cell line. Environ. Toxicol. Chem..

[31] Dalecka B., Juhna T., Rajarao G.K. (2020). Constructive use of filamentous fungi to remove pharmaceutical substances from wastewater. J. Water Process Eng..

[32] Amasha M., Baalbaki A., Ghauch A. (2018). A comparative study of the common persulfate activation techniques for the complete degradation of an NSAID: The case of ketoprofen. Chem. Eng. J..

[33] Kanakaraju D., Glass B.D., Oelgemöller M. (2018). Advanced oxidation process-mediated removal of pharmaceuticals from water: A review. J. Environ. Manag..

[34] Zhen H., Liu J., Zhang T. (2022). The Effect of UV degradation of ketoprofen and its influencing factors. J. Phys. Conf. Ser..

[35] Acosta I., Moctezuma E., López de la O K., Leyva E., Zermeno B. (2022). Photocatalytic degradation of high concentration aqueous solutions of ketoprofen: Adsorption, reaction kinetic and product studies. Top. Catal..

[36] Illés E., Takács E., Dombi A., Gajda-Schrantz K., Gonter K., Wojnárovits L. (2012). Radiation induced degradation of ketoprofen in dilute aqueous solution. Radiat. Phys. Chem..

[37] Paparo R., Viscovo A., Trifuoggi M., Di Serio M., Russo V. (2024). Ketoprofen photodegradation kinetics promoted by TiO_2_. ChemEngineering.

[38] Gao Y.Q., Zhou J.Q., Rao Y.Y., Ning H., Zhang J., Shi J., Gao N.Y. (2022). Comparative study of degradation of ketoprofen and paracetamol by ultrasonic irradiation: Mechanism, toxicity and DBP formation. Ultrason. Sonochem..

[39] Martínez C., Vilariño S., Fernández M.I., Faria J., Canle L.M., Santaballa J.A. (2013). Mechanism of degradation of ketoprofen by heterogeneous photocatalysis in aqueous solution. Appl. Catal. B Environ..

[40] de Melo Santos M.M., da Silva T.D., de Lucena A.L.A., Napoleão D.C., Duarte M.M.M.B. (2020). Degradation of ketoprofen, tenoxicam, and meloxicam drugs by photo-assisted peroxidation and photo-fenton processes: Identification of intermediates and toxicity study. Water Air Soil Pollut..

[41] Feng L., Oturan N., van Hullebusch E.D., Esposito G., Oturan M.A. (2014). Degradation of anti-inflammatory drug ketoprofen by electro-oxidation: Comparison of electro-Fenton and anodic oxidation processes. Environ. Sci. Pollut. Res..

[42] Song W., Lu H., Li Q., Wang X., Fu Z., Zhou J. (2023). Aerobic degradation of ketoprofen by marine consortia: Fenton-like reaction and degradation pathway. Sci. Total Environ..

[43] Park J.S., Her N.G., Yoon Y. (2011). Sonochemical degradation of chlorinated phenolic compounds in water: Effects of physicochemical properties of the compounds on degradation. Water Air Soil Pollut..

[44] Quintana J., Weiss S., Reemtsma T. (2005). Pathways and metabolites of microbial degradation of selected acidic pharmaceutical and their occurrence in municipal wastewater treated by a membrane bioreactor. Water Res..

[45] Ismail M.M., Essam T.M., Ragab Y.M., Mourad F.E. (2016). Biodegradation of ketoprofen using a microalgal–bacterial consortium. Biotechnol. Lett..

[46] Can-Ubando L.C., Ángeles-de Paz G., Isaac-Olivé K., Aranda E., Sánchez-Reyes A., Sandoval-Trujillo H., Ramírez-Durán N. (2025). Degradation of diclofenac and ketoprofen by *Bacillus paralicheniformis* HAS-1, strain isolated from a soda lake sediment. Water Air Soil Pollut..

[47] Marco-Urrea E., Pérez-Trujillo M., Cruz-Morató C., Caminal G., Vicent T. (2010). White-rot fungus-mediated degradation of the analgesic ketoprofen and identification of intermediates by HPLC–DAD–MS and NMR. Chemosphere.

[48] Apriceno A., Astolfi M.L., Girelli A.M., Scuto F.R. (2019). A new laccase-mediator system facing the biodegradation challenge: Insight into the NSAIDs removal. Chemosphere.

[49] Al-sareji O.J., Meiczinger M., Salman J.M., Al-Juboori R.A., Hashim K.S., Somogyi V., Jakab M. (2023). Ketoprofen and aspirin removal by laccase immobilized on date stones. Chemosphere.

[50] Cuklev F., Fick J., Cvijovic M., Kristiansson E., Förlin L., Larsson D.G.J. (2012). Does ketoprofen or diclofenac pose the lowest risk to fish?. J. Hazard. Mater..

[51] United Nations (2011). Globally Harmonized System of Classification and Labelling of Chemicals (GHS).

[52] Świacka K., Smolarz K., Maculewicz J., Michnowska A., Caban M. (2021). Exposure of *Mytilus trossulus* to diclofenac and 4′-hydroxydiclofenac: Uptake, bioconcentration and mass balance for the evaluation of their environmental fate. Sci. Total Environ..

[53] Naidoo V., Wolter K., Cromarty D., Diekmann M., Duncan N., Meharg A.A., Taggart M.A., Venter L., Cuthbert R. (2010). Toxicity of non-steroidal anti-inflammatory drugs to *Gyps* vultures: A new threat from ketoprofen. Biol. Lett..

[54] Minguez L., Pedelucq J., Farcy E., Ballandonne C., Budziński H., Halm-Lemeille M.P. (2014). Toxicities of 48 pharmaceuticals and their freshwater and marine environmental assessment in northwestern France. Environ. Sci. Pollut. Res..

[55] Vargas E.L.G., de Almeida F.A., de Freitas L.L., Pinto U.M., Vanetti M.C.D. (2021). Plant compounds and nonsteroidal anti-inflammatory drugs interfere with quorum sensing in *Chromobacterium violaceum*. Arch. Microbiol..

[56] Cycoń M., Borymski S., Żołnierczyk B., Piotrowska-Seget Z. (2016). Variable effects of non-steroidal anti-inflammatory drugs (NSAIDs) on selected biochemical processes mediated by soil microorganisms. Front. Microbiol..

[57] Pawłowska B., Telesiński A., Sysa M., Godela A., Ščurek R., Biczak R. (2023). Ibuprofen and ketoprofen—Inert drugs or potential environmental hazard?. Sustainability.

[58] Matejczyk M., Rosochacki S.J., Jabłońska-Trypuć A. (2016). Monitoring of pharmaceutical residues of non-steroidal drugs with use of *Escherichia coli*-GFP biosensors. Ecol. Chem. Eng. A.

[59] Marchlewicz A., Guzik U., Hupert-Kocurek K., Nowak A., Wilczyńska S., Wojcieszyńska D. (2017). Toxicity and biodegradation of ibuprofen by *Bacillus thuringiensis* B1(2015b). Environ. Sci. Pollut. Res..

[60] Guzik U., Wojcieszyńska D., Kumar A., Sharma S. (2019). Biodegradation of non-steroidal anti-inflammatory drugs and their influence on soil microorganisms. Microbes and Enzymes in Soil Health and Bioremediation.

[61] Ionescu L., Gheorghe S., Mitru D., Stoica C., Banciu A.R., Mihalache M., Nita-Lazar M. (2020). Evaluating the ecotoxicity of different pharmaceuticals using *Aliivibrio fischeri* bioassays. Rom. J. Ecol. Environ. Chem..

[62] Miller M.B., Bassler B.L. (2001). Quorum sensing in bacteria. Annu. Rev. Microbiol..

[63] Tajani A.S., Jangi E., Davodi M., Golmakaniyoon S., Ghodsi R., Soheili V., Fazly Bazzaz B.S. (2021). Anti-quorum sensing potential of ketoprofen and its derivatives against *Pseudomonas aeruginosa*: Insights to in silico and in vitro studies. Arch. Microbiol..

[64] Bownik A., Jasieczek M., Kosztowny E. (2020). Ketoprofen affects swimming behavior and impairs physiological endpoints of *Daphnia magna*. Sci. Total Environ..

[65] Alkimin G.D., Soares A.M.V.M., Barata C., Nunes B. (2020). Evaluation of ketoprofen toxicity in two freshwater species: Effects on biochemical, physiological and population endpoints. Environ. Pollut..

[66] Mezzelani M., Gorbi S., Fattorini D., d’Errico G., Consolandi G., Milan M., Bargelloni L., Regoli F. (2018). Long-term exposure of Mytilus galloprovincialis to diclofenac, ibuprofen and ketoprofen: Insights into bioavailability, biomarkers and transcriptomic changes. Chemosphere.

[67] Żbikowska E., Lombardo P., Żbikowski J., Jabłońska G., Marszewska A., Cichy A. (2017). Ketoprofen-induced inhibition of symptoms of behavioural fever observed in wintering *Planorbarius corneus* (L.) (Gastropoda: Planorbidae). J. Molluscan Stud..

[68] Manjarrés-López D.P., Peña-Herrera J.M., Benejam L., Montemurro N., Pérez S. (2023). Assessment of wastewater-borne pharmaceuticals in tissues and body fluids from riverine fish. Environ. Pollut..

[69] Mello F.V., Cunha S.C., Fogaça F.H.S., Alonso M.B., Torres J.P.M., Fernandes J.O. (2022). Occurrence of pharmaceuticals in seafood from two Brazilian coastal areas: Implication for human risk assessment. Sci. Total Environ..

[70] Pashaei R., Dzingelevičienė R., Abbasi S., Szultka-Młyńska M., Buszewski B. (2022). Determination of 15 human pharmaceutical residues in fish and shrimp tissues by high-performance liquid chromatography-tandem mass spectrometry. Environ. Monit. Assess..

[71] Rangasamy B., Hemalatha D., Shobana C., Nataraj B., Ramesh M. (2018). Developmental toxicity and biological responses of zebrafish (*Danio rerio*) exposed to anti-inflammatory drug ketoprofen. Chemosphere.

[72] Praskova E., Voslarova E., Siroka Z., Macova S., Plhalova L., Bedanova I., Marsalek P., Pistekova V., Svobodova Z. (2011). Comparison of acute toxicity of ketoprofen to juvenile and embryonic stages of *Danio rerio*. Neuro Endocrinol. Lett..

[73] Madesh S., Sudhakaran G., Murugan R., Almutairi M.H., Almutairi B.O., Kathiravan M.K., Arockiaraj J. (2024). Parental (F0) exposure to cadmium and ketoprofen induces developmental deformities in offspring (F1): A transgenerational toxicity assessment in zebrafish model. Sci. Total Environ..

[74] Wang H., Jin M., Xu L., Xi H., Wang B., Du S., Liu H., Wen Y. (2020). Effects of ketoprofen on rice seedlings: Insights from photosynthesis, antioxidative stress, gene expression patterns, and integrated biomarker response analysis. Environ. Pollut..

